# Syphilitic encephalitis - a rare disease and a possible differential diagnosis of herpetic encephalitis

**DOI:** 10.1186/1471-2334-14-S7-P97

**Published:** 2014-10-15

**Authors:** Mihaela Ionică, Magdalena Vasile, Șerban Benea, Virgil Ionescu, Elisabeta Benea

**Affiliations:** 1 National Institute for Infectious Diseases "Prof. Dr. Matei Balş", Bucharest, Romania

## Background

Syphilitic encephalitis is an atypical presentation form of central neural system infection by *Treponema pallidum*. There are only few case-reports in medical literature.

## Case report

We present the case of a 41 year-old male, diagnosed with syphilitic encephalitis, in whom cerebral magnetic resonance imaging demonstrated preponderant involvement of bilateral temporal lobes, for this point of view raising differential diagnostic concerns with Herpes virus encephalitis. We also identified multiple encephalitis foci: hippocampus, lentiform nucleus, left thalamus, left midbrain, and bilateral occipital. The detection of herpes simplex virus DNA by PCR in CSF obtained at admission was negative. Furthermore, the subacute onset as a maniacal syndrome delayed the diagnostic, the patient being initially treated as psychiatric disorder in a Psychiatric Unit.

## Conclusion

Although the clinical and imagistic evolution was favorable under specific optimal therapy, the patient had some neuropsychiatric sequels and is currently enrolled in a medical recovery treatment.

## Consent

Written informed consent was obtained from the patient for publication of this Case report and any accompanying images. A copy of the written consent is available for review by the Editor of this journal.

